# Supplementary material for: Ecogenomics reveals viral communities across the Challenger Deep oceanic trench
Source: Commun Biol. 2022 Oct 4;5:1055. doi: 10.1038/s42003-022-04027-y (PMC9529941; doi:10.1038/s42003-022-04027-y)
Supplement: Supplementary file 2 — Supplementary Information [file 42003_2022_4027_MOESM2_ESM.pdf]

Supplementary Information

Ecogenomics reveals viral communities across the Challenger Deep oceanic trench

Ying-Li Zhou[1,2, #], Paraskevi Mara[3, #], Dean Vik[4], Virginia P. Edgcomb[3], Matthew B. Sullivan[4,5], Yong Wang[1,6, *]

[1] Institute of Deep-Sea Science and Engineering, Chinese Academy of Sciences, Sanya, Hainan, China

[2] University of the Chinese Academy of Sciences, Beijing, China

[3] Department of Geology and Geophysics, Woods Hole Oceanographic Institution, Woods Hole, MA, USA

[4] Department of Microbiology and Center of Microbiome Science, The Ohio State University, Columbus, OH, USA

[5] Department of Civil, Environmental and Geodetic Engineering, The Ohio State University, Columbus, OH, USA

[6] Institute for Ocean Engineering, Shenzhen International Graduate School, Tsinghua University, Shenzhen, China

# These authors contributed equally

* Yong Wang

Email: wangyong@sz.tsinghua.edu.cn

Supplementary Information includes:

Supplementary Figures 1-10.

Supplementary Tables 1 and 2.

Legends for Supplementary Data 1-11. Supplementary Data 1-11 are provided in separate excel files.

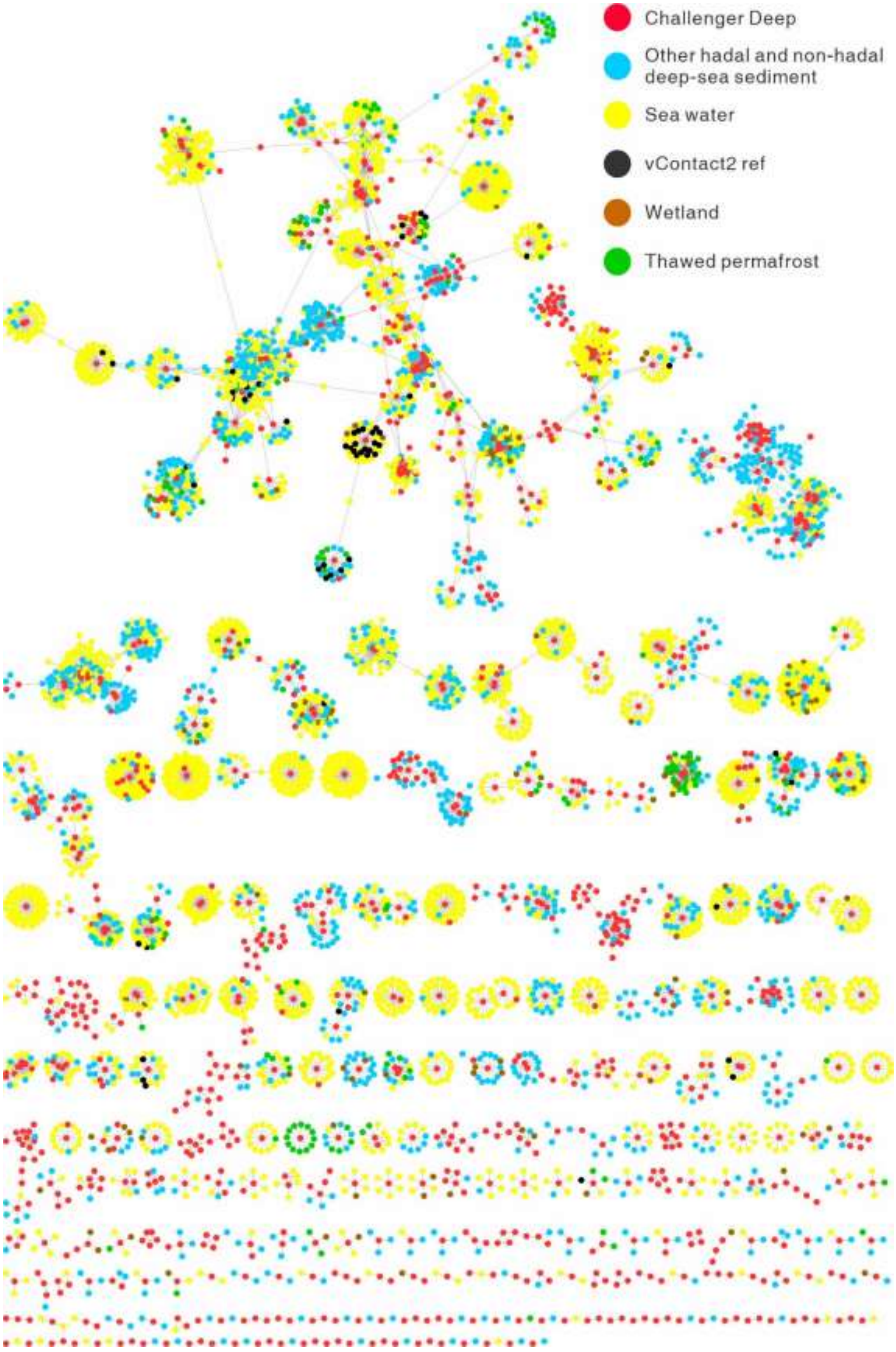
Challenger Deep
Other hadal and non-hadal deep-sea sediment
Sea water
vContact2 ref
Wetland
Thawed permafrost

Supplementary Fig. 1. Protein-sharing network between viral contigs from Challenger Deep (n = 1 628), other hadal and non-hadal deep-sea (n = 2 885) and wetland sediments (n = 1 212), Global Ocean Virome 2.0 (n = 195 728), thawed permafrost soils (n=1 907), and vContact2 RefSeq prokaryotic viral genomes (n = 2 010). Nodes (circles) represent viral contigs. Shared edges (lines) indicate shared protein content. Well-supported viral clusters containing CD vOTUs were shown in this figure.

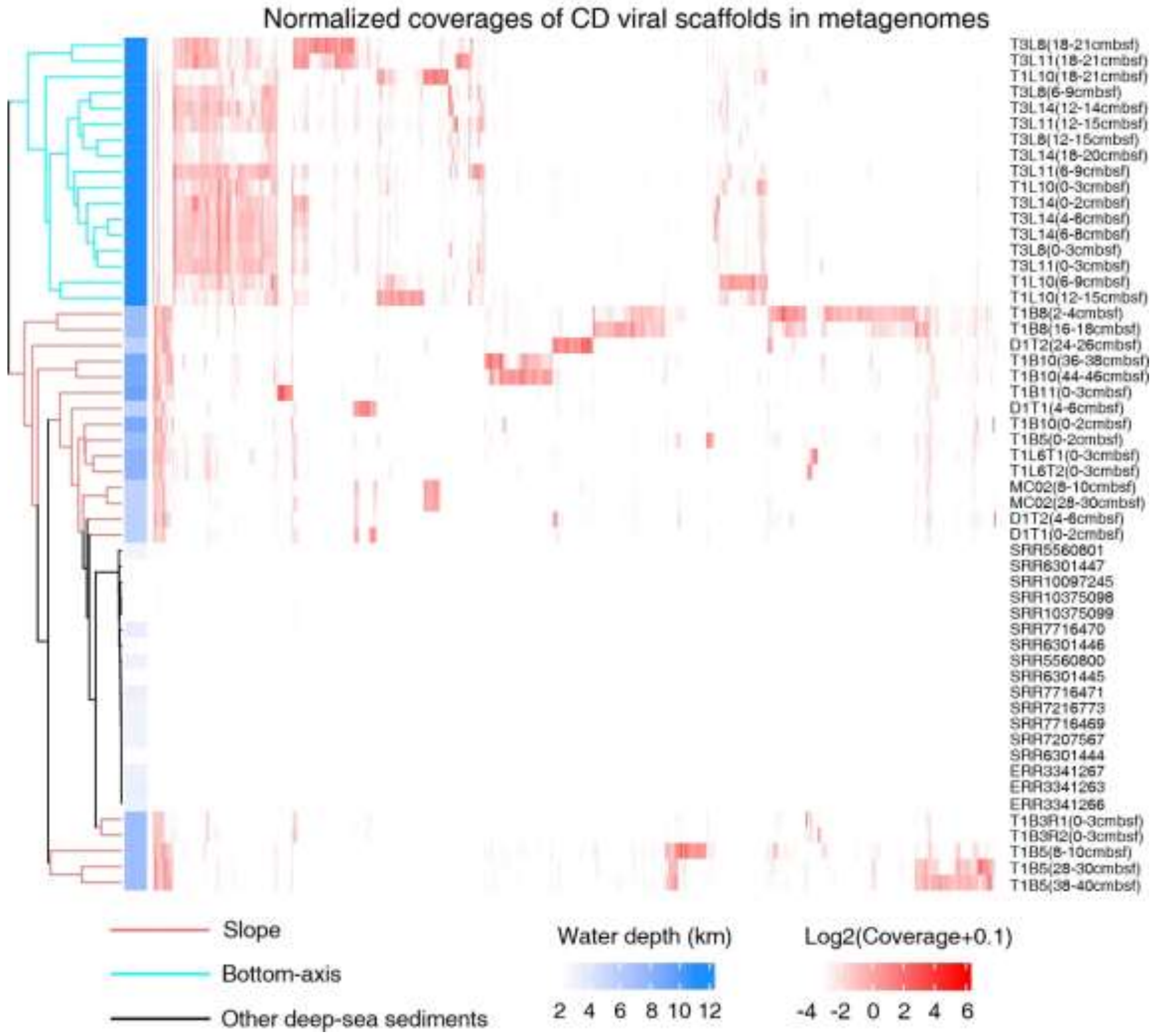

Supplementary Fig. 2. Normalized coverages of viral populations (species level) in CD and reference deep-sea metagenomes. Each row represents an individual CD metagenome, while each column represents an individual CD viral population. The normalized relative abundance values (log2-transformed) are shown in color scale. Red, blue and black lines represent the hierarchical clustering of slope, bottom-axis and other deep-sea sediments, respectively.

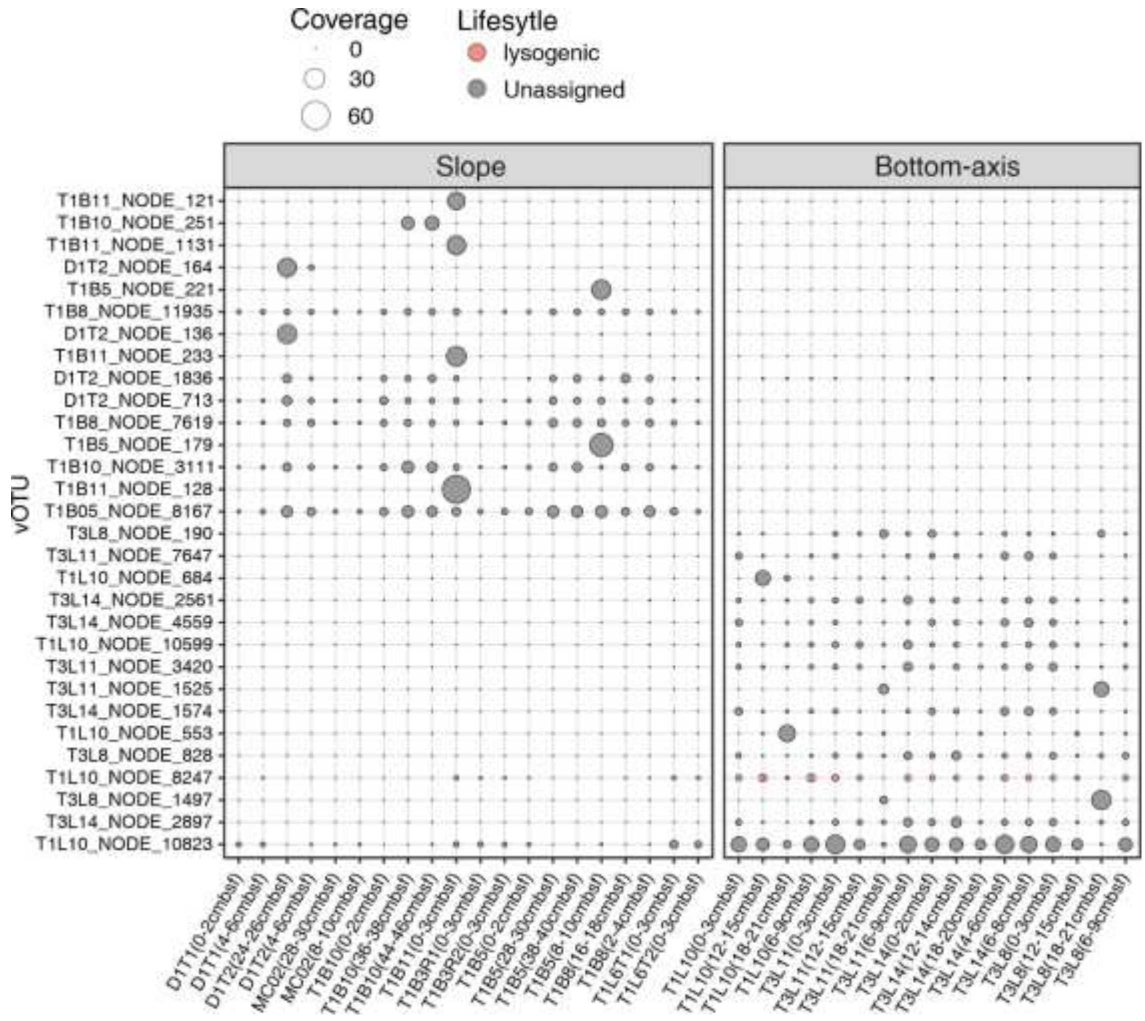

Supplementary Fig. 3. Relative abundance of the 15 most abundant viral populations for slope and bottom-axis samples. The bubbles indicate the normalized coverage (coverage per giga base), and the predicted lifestyle of viruses for each sample.

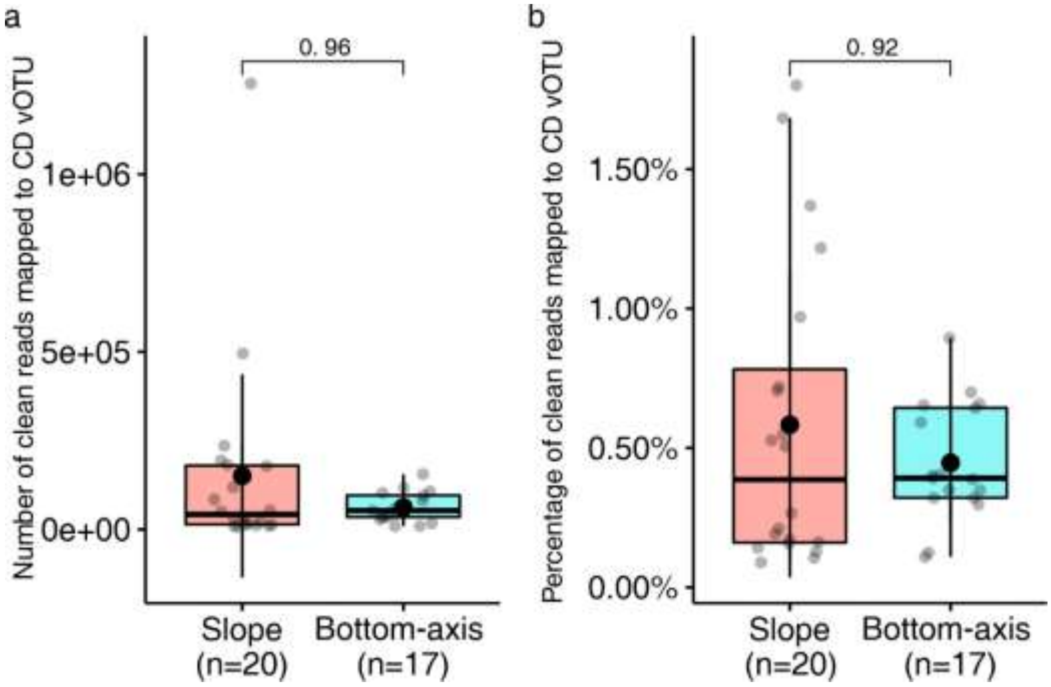

Supplementary Fig. 4. Sequencing depth of CD viromes in number of total paired reads per metagenome. (a) The number of paired-end reads mapped to CD vOTUs. (b) Percentage of paired-end reads mapped to CD vOTUs in metagenomes. *p*-value was estimated using the two-sided Wilcoxon test. For boxplots, centre line indicates median, bounds of box indicate 25th and 75th percentiles, and whiskers indicate minimum and maximum.

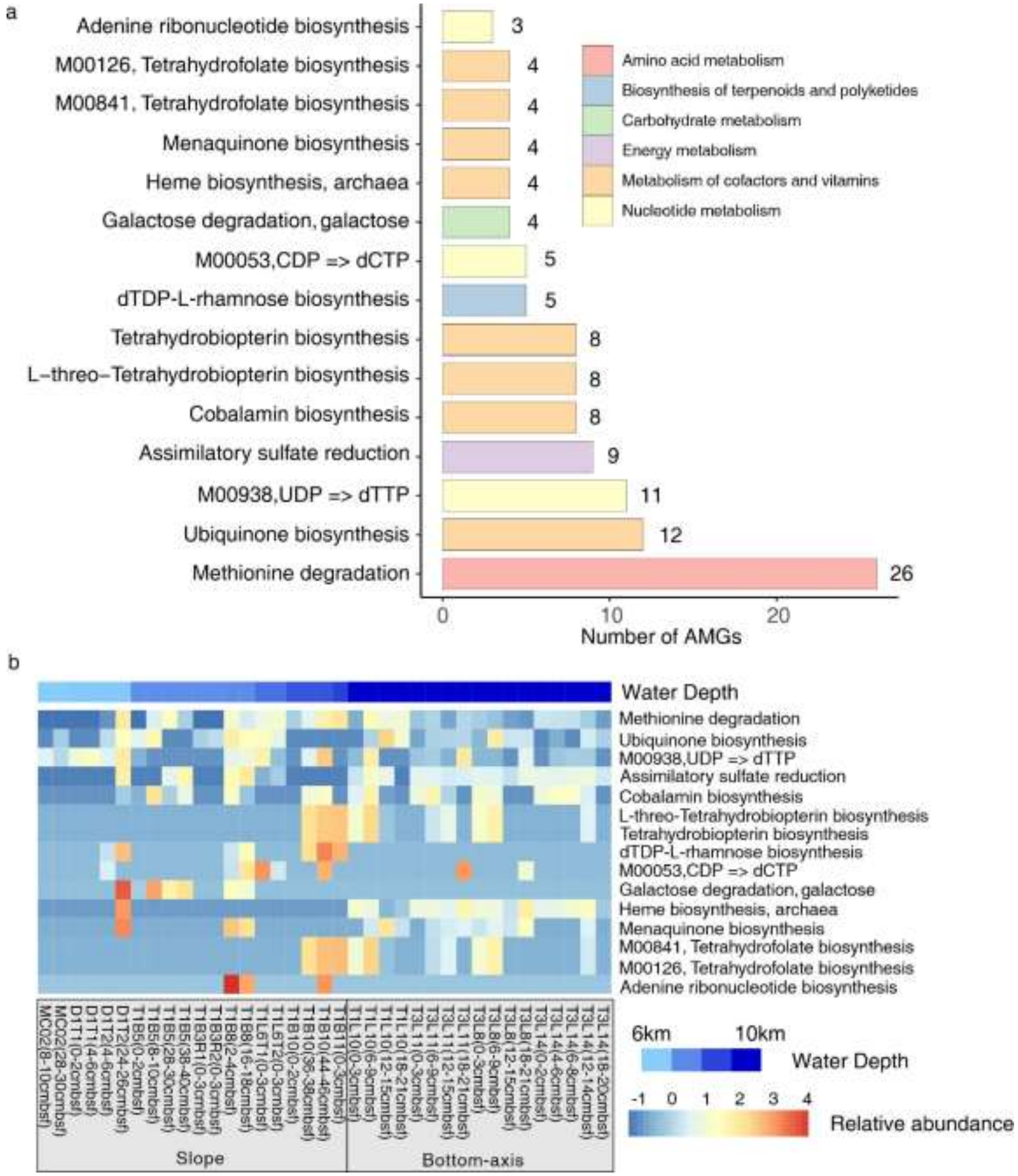

Supplementary Fig. 5 Overview of putative AMGs in CD viral communities. (**a**) Number of putative AMGs involved ranked by AMGs prevalence in CD virome (top fifteen shown). (**b**) Abundance of viruses that have genes belonging to the fifteen most prevalent AMGs identified in CD sediment viromes. The genes identified in various KEGG-described metabolisms are listed in Supplementary Data 9. Relative abundances were log2 transformed and normalized with a z-score method based on normalized vOTUs coverage.

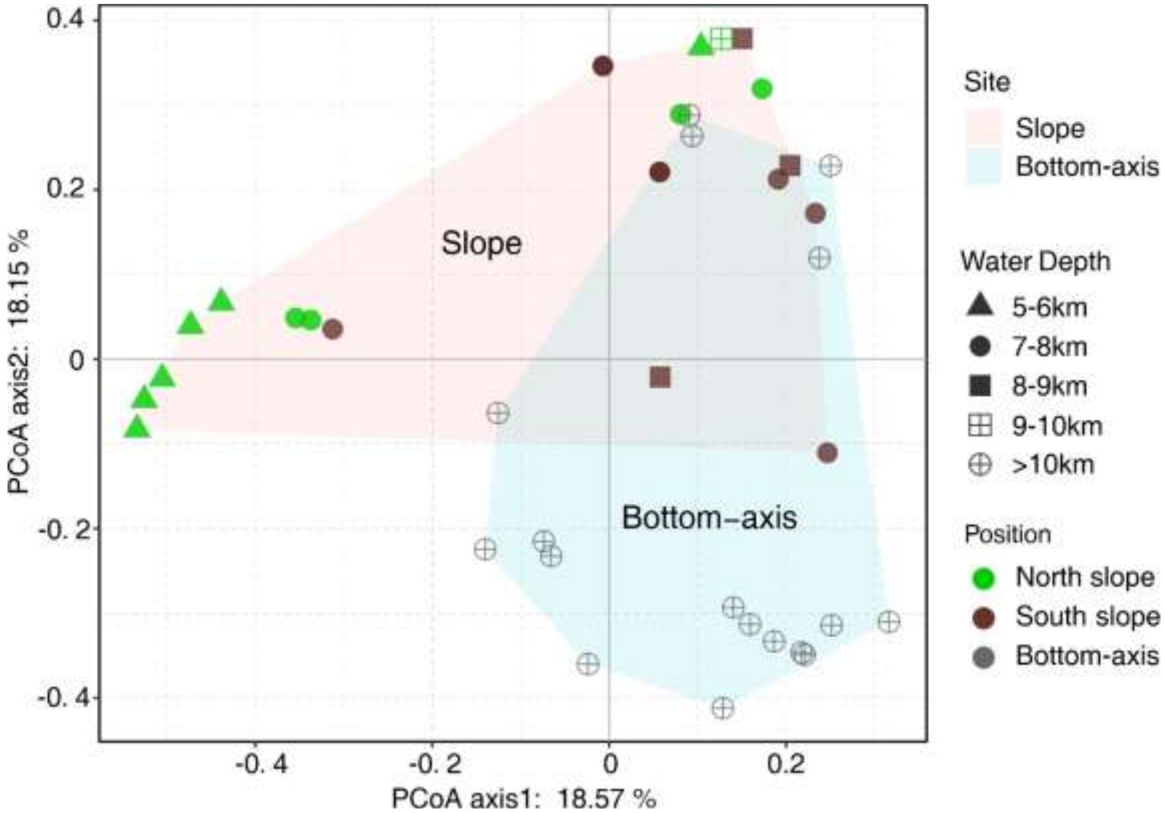

Supplementary Fig. 6. Bray-Curtis dissimilarity principal coordinate analyses (PCoA) of the viral AMGs. The pink and blue areas covered slope and bottom-axis samples, respectively.

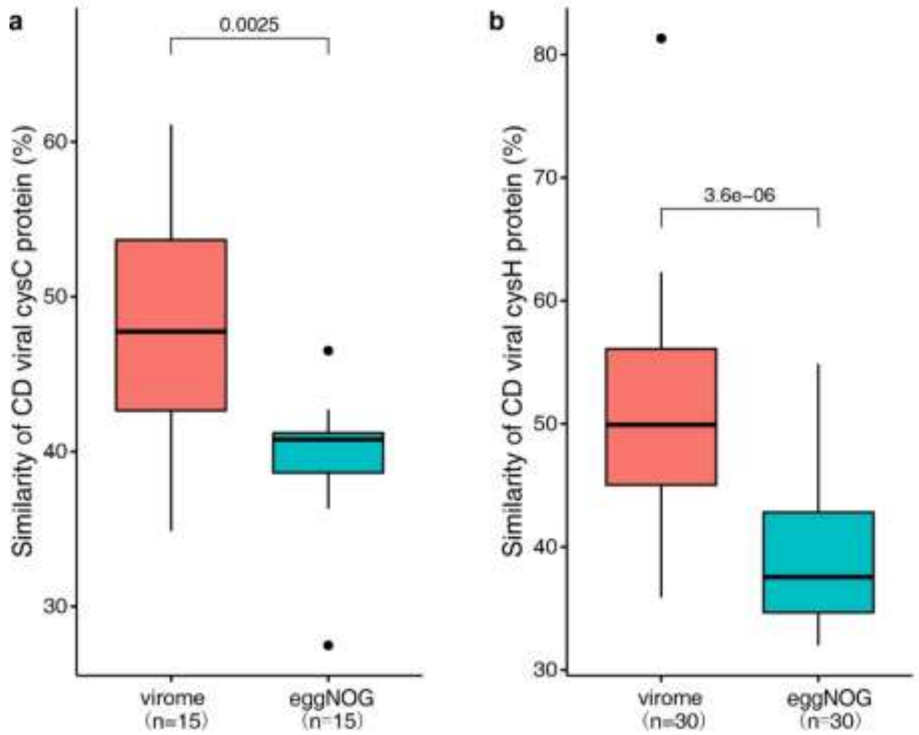

Supplementary Fig. 7. Similarity of each Cys protein (CysC/CysH) with the five closest homologues recruited from public viromes and the eggNOG database, respectively. Three CysC (**a**) and six CysH proteins (**b**) from CD viral contigs. Boxplots represent the median with 25th and 75th percentiles, and whiskers show the 1.5 interquartile range.

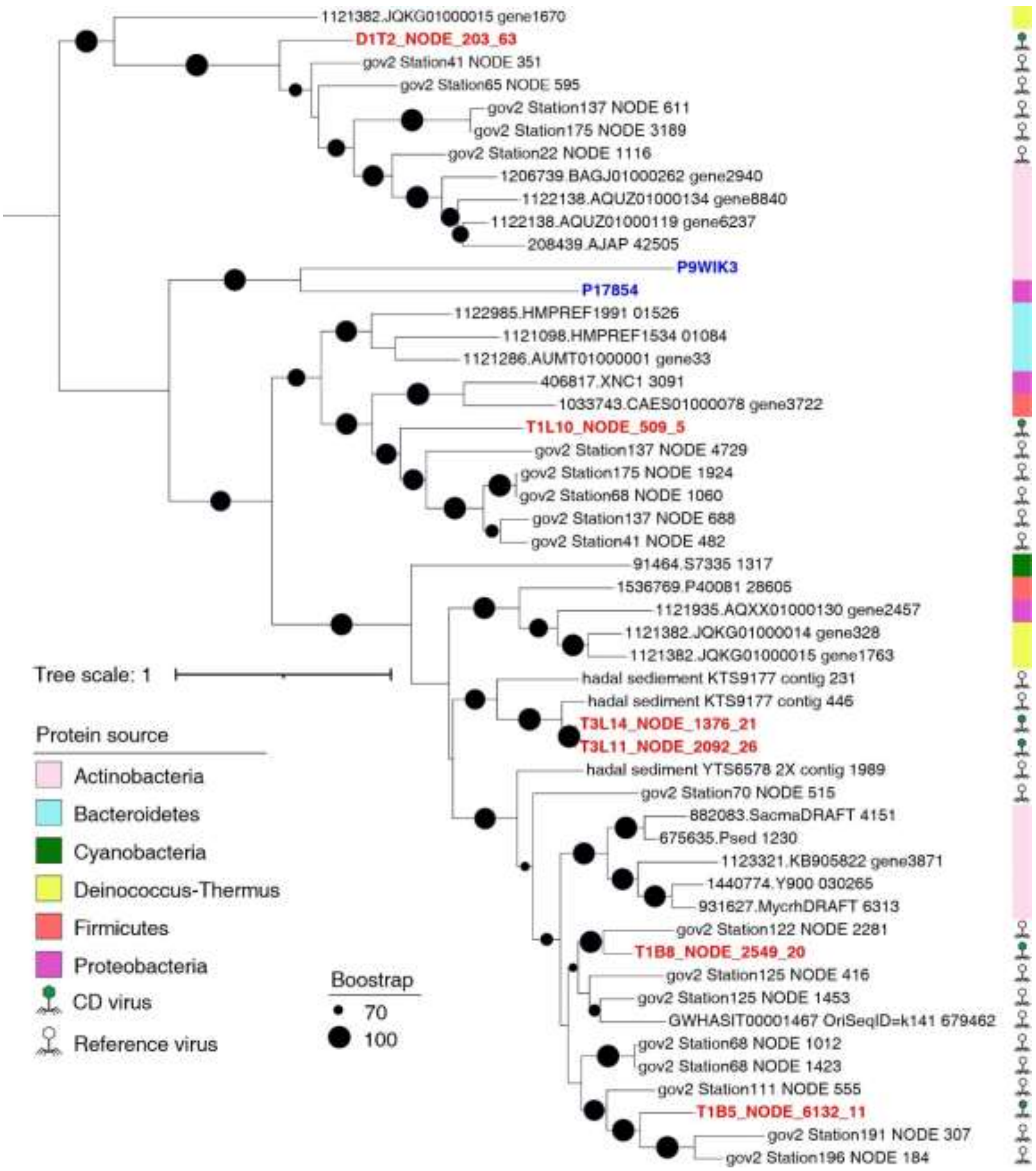

Supplementary Fig. 8. Maximum-likelihood phylogenetic tree between the identified CD CysH proteins (this study) and the CysH proteins found in eggNOG database (V5.0). The tree is rooted in the mid-point. Two CysH homologs from Uniport database are in blue, and indicate protein function based on experimental evidence. Bootstrap values (1 000 replicates) ≥ 70% are indicated at nodes.

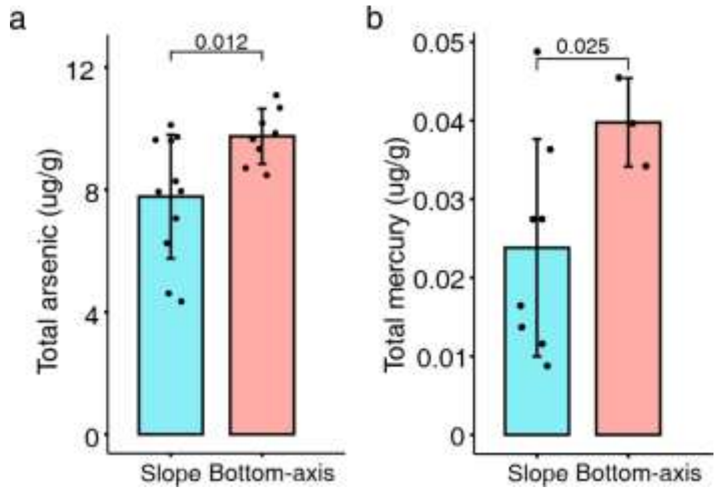

Supplementary Fig. 9. Total arsenic and mercury concentrations (μg g^{-1} of dry sediment) in 13 CD sites (5 400-10 911 m depth). *p* values were estimated using two-sided Wilcoxon test for pairwise comparisons. CD sediments were analyzed for total arsenic and mercury using Atomic Fluorescence Spectrometry. Bar graphs identify mean and standard error bars.

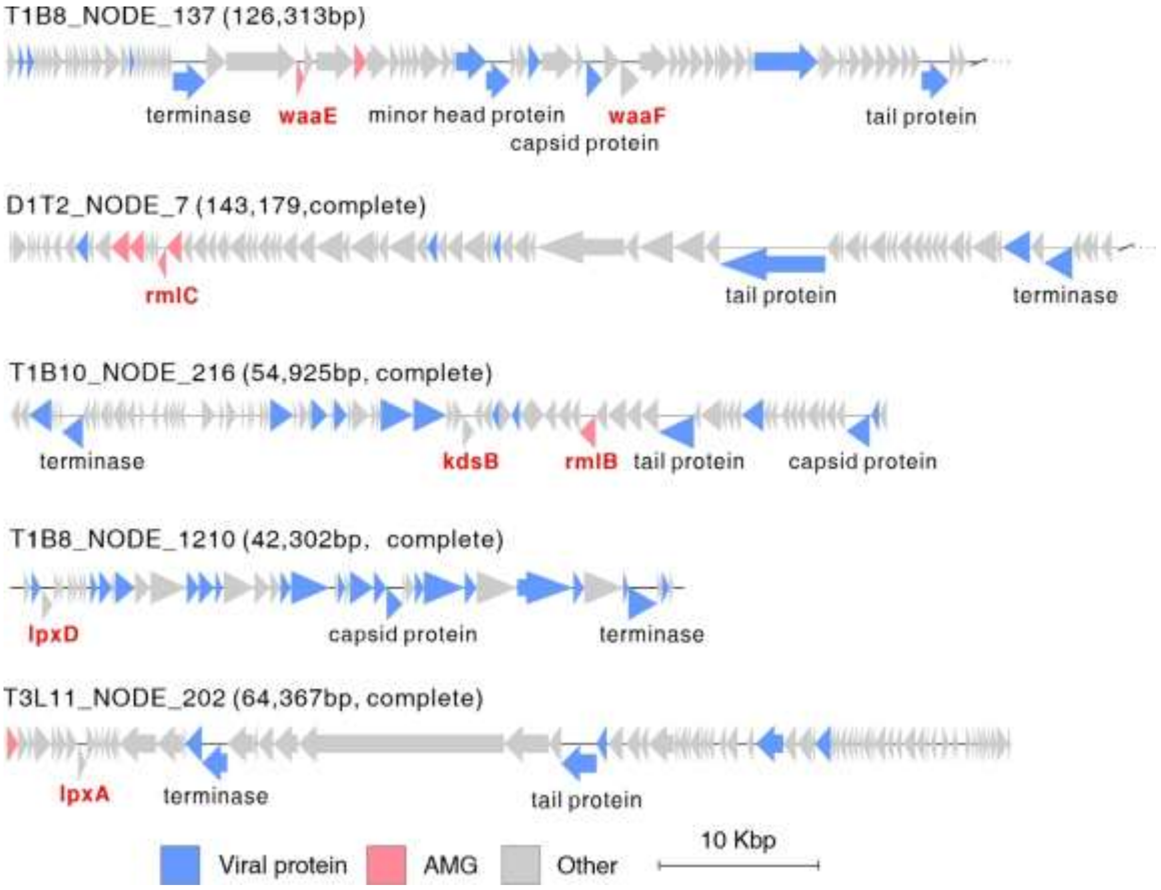

Supplementary Fig. 10. Genome maps of the viral contigs that contain LPS and dTDP-L-rhamnose synthetic genes. The maps indicate the positions of these genes in the viromes, and the upstream and/or downstream phage genes. Viral genes were annotated by CheckV and VIBRANT. AMGs, non-phage-like and uncharacterized genes are shown in blue, pink and gray, respectively. Detailed functional annotation of the viral scaffolds can be found in Supplementary Data 8.

Supplementary Table 1. Sampling sites and sampling methods used for collecting the sediment samples in CD

Cruise	Sample station	Sampling method	Latitude [North]	Longitude [East]	Water Depth [m]	Position in trench	Layers for metagenome [cmbsf]	Layers for metatranscriptome [cmbsf]
DY37II	MC02	push core (submersible)	11.764	141.976	5 400	slope	8-10; 28-30	\
DY37II	D1T1#	push core (submersible)	11.801	142.117	5 533	slope	0-2; 4-6	\
DY37II	D1T2#	push core (submersible)	11.801	142.117	5 533	slope	4-6; 24-26	\
TS01	T1B5	box core (hydrographic cable)	10.924	141.799	7 061	slope	0-2; 8-10; 28-30; 38-40	\
TS01	T1B3	box core (hydrographic cable)	11.553	141.873	7 082	slope	0-3	\
TS01	T1B8	box core (hydrographic cable)	11.602	142.228	7 143	slope	2-4; 16-18	\
TS01	T1L6	push core (lander)	11.091	142.073	7 850	slope	0-2 (two push cores)	\
TS01	T1B10	box core (hydrographic cable)	11.195	141.812	8 638	slope	0-2; 36-38; 44-46	\
TS01	T1B11	box core (hydrographic cable)	11.228	141.689	9 150	slope	0-3	\
TS03	T3L11	push core (lander)	11.325	142.191	10 908	bottom-axis	0-3; 6-9; 12-15; 18-21	6-9; 12-15; 18-21
TS03	T3L8	push core (lander)	11.327	142.194	10 909	bottom-axis	0-3; 6-9; 12-15; 18-21	\
TS03	T3L14	push core (lander)	11.325	142.189	10 911	bottom-axis	0-2; 4-6; 6-8; 12-14; 18-20	\
TS01	T1L10	push core (lander)	11.328	142.202	10 900	bottom-axis	0-3; 6-9; 12-15; 18-21	\

#: sediment samples from two slope sites (~ 100m apart) collected in a single dive of the submersible “Jiaolong”.

Supplementary Table 2. List of marine viruses related datasets

Environment source	Ocean virome dataset	Ocean and sea regions (number of samples)	Total number of viromes	Sequencing technology	Data size (Gb)	Reference
Ocean water	The Marine Viromes of Four Oceanic Regions	Arctic Ocean (56; Canadian Arctic and Chukchi Sea), North Atlantic Ocean (42; Sargasso Sea (1), Gulf of Mexico (41)), North Pacific Ocean (85; British Columbia coastal waters)	4 viromes from 183 integrative samples	Life Sciences 454 pyrosequencing	0.181	[1]
	Temperate and subtropical seawater	Saanich Inlet (11), Strait of Georgia (85), Gulf of Mexico (46)	142	Roche 454 Titanium	1.9 *	[2]
	Tara Oceans Viromes (TOV)	Mediterranean Sea (7), Red Sea (5), Indian Ocean (16), North Atlantic Ocean (0), South Atlantic Ocean (12), Southern Ocean (1), North Pacific Ocean (2), South Pacific Ocean (0)	43	Illumina HiSeq 2000	218 *	[3]
	Global Ocean Viromes (GOV)	Mediterranean Sea (8), Red Sea (6), Indian Ocean (27), North Atlantic Ocean (4), South Atlantic Ocean (19), Southern Ocean (4), North Pacific Ocean (15), South Pacific Ocean (21)	104	Illumina HiSeq 2000	925	[4]
	Baltic Sea Viromes	Baltic Sea (11)	21	Roche 454 pyrosequencing	NA	[5]
	Global Ocean Viromes 2.0 (GOV 2.0)[#]	Arctic Ocean (38), North Atlantic Drift Province (3; station 155), all GOV samples (104)	145	Illumina HiSeq 2000	3950	[6]
Hadal and non-hadal deep-sea sediment	Hadal sediment viromes	Izu-Ogasawara Trench (1), Mariana Trench (1)	2	454 GS FLX Titanium	0.029	[7]
	Arctic Ocean metagenome	Arctic Ocean: Southern Knipovich Ridge (3) Gakkel Ridge, Loki's castle (1)	4	Illumina HiSeq 2500	244	[8]
	Southwest Indian Ocean bulk metagenome	Southwest Indian Ocean (6)	6	Illumina Hiseq-2500	373	[9]
	Cold seep bulk metagenome[#]	Haakon Mosby mud volcano (6); Eastern North Pacific ODP site 1244 (7); Mediterranean Sea-Amon mud volcano (1); Santa Monica Mounds (1); Eastern Gulf of Mexico (3); Scotian Basin (8); and Western Gulf of Mexico (7)	40	Illumina (MiSeq, NextSeq 500, HiSeq 1500, HiSeq 2500, HiSeq 1000	411	[10]

	Hadal sediment bulk metagenome[#]	Marina Trench (2), Kermadec Trench (2), Yap Trench (3)	7	Illumina HiSeq 2500, HiSeq X Ten	250	[11]
	Challenger Deep bulk metagenome	Slope (20) and bottom-axis (17) sites of Challenger Deep in Mariana Trench	37 metagenomes and 3 metatranscriptomes	Illumina MiSeq, Illumina HiSeq 2500,	283	This study

* Estimated by multiplying the number of reads by the average read length

Shows datasets used for comparisons in this study

Supplementary references

1. Angly FE, *et al.* The marine viromes of four oceanic regions. *PLOS Biol* **4**, 2121-2131 (2006).

2. Labonté JM, Suttle CA. Previously unknown and highly divergent ssDNA viruses populate the oceans. *ISME J* **7**, 2169-2177 (2013).

3. Brum JR, *et al.* Patterns and ecological drivers of ocean viral communities. *Science* **348**, 1261498 (2015).

4. Roux S, *et al.* Ecogenomics and potential biogeochemical impacts of globally abundant ocean viruses. *Nature* **537**, 689-693 (2016).

5. Allen LZ, *et al.* The Baltic sea virome: diversity and transcriptional activity of DNA and RNA viruses. *mSystems* **2**, e00125-00116 (2017).

6. Gregory AC, *et al.* Marine DNA viral macro- and microdiversity from pole to pole. *Cell* **177**, 1109–1123 (2019).

7. Yoshida M, Takaki Y, Eitoku M, Nunoura T, Takai K. Metagenomic analysis of viral communities in (hado)pelagic sediments. *PLoS One* **8**, e57271 (2013).

8. Bäckström D, *et al.* Virus genomes from deep sea sediments expand the ocean megavirome and support independent origins of viral gigantism. *mBio* **10**, e02497-02418 (2019).

9. Zheng X, *et al.* Extraordinary diversity of viruses in deep-sea sediments as revealed by metagenomics without prior virion separation. *Environ Microbiol* **23**, 728-743 (2021).

10. Li Z, *et al.* Deep sea sediments associated with cold seeps are a subsurface reservoir of viral diversity. *ISME J* **15**, 2366–2378 (2021).

11. Jian H, *et al.* Diversity and distribution of viruses inhabiting the deepest ocean on Earth. *ISME J* **15**, 3094–3110 (2021).
