# Supplementary material for: Ecogenomics reveals viral communities across the Challenger Deep oceanic trench
Source: Commun Biol. 2022 Oct 4;5:1055. doi: 10.1038/s42003-022-04027-y (PMC9529941; doi:10.1038/s42003-022-04027-y)
Supplement: Supplementary file 3 — Description of Additional Supplementary Files [file 42003_2022_4027_MOESM3_ESM.pdf]

Description of Additional Supplementary Files

File Name: Supplementary Data 1

Description: Overview of the metagenomes and metatranscriptomes generated from the slope and bottom-axis CD sediments.

File Name: Supplementary Data 2

Description: Overview of the criteria for What the Phage to identify viral contigs by the raw output results of each tool.

File Name: Supplementary Data 3

Description: Quality and taxonomy of CD viral scaffolds.

File Name: Supplementary Data 4

Description: Different percentages of genes mapped in each virus using metatranscriptome reads to determine potentially activate viruses.

File Name: Supplementary Data 5

Description: Relative abundance of CD vOTUs (species level) in metagenomes and CD vOTUs mapped reads in metatranscriptomes.

File Name: Supplementary Data 6

Description: Relative abundance of CD viruses at family level.

File Name: Supplementary Data 7

Description: Predicted prokaryotic hosts of CD vOTUs. Sheet1: Identifications of hosts, Sheet2: Relative abundance of hosts of CD vOTUs

File Name: Supplementary Data 8

Description: Gene annotations of in CD vOTUs using CheckV, eggNOG, VIBRANT and DRAM-v. Sheet1: Functional annotations of 1 628 CD vOTUs, Sheet2: manually curated vOTUs for analysis of functional metabolic genes (AMGs; see also main text). Sheet3: CD vOTUs removed from AMGs analysis.

File Name: Supplementary Data 9

Description: Summary of putative auxiliary metabolic genes (AMGs) detected in CD vOTUs using KEGG.

File Name: Supplementary Data 10

Description: Closest homologs of CD AMGs in the eggNOG database and other viromes (viromes from marine water and deep-sea sediments). Sheet1: Homologs in the eggNOG database. Sheet2: Homologs in other viromes.

File Name: Supplementary Data 11

Description: Source data for figures.
