# Supplementary material for: Ecogenomics reveals viral communities across the Challenger Deep oceanic trench
Source: Commun Biol. 2022 Oct 4;5:1055. doi: 10.1038/s42003-022-04027-y (PMC9529941; doi:10.1038/s42003-022-04027-y)
Supplement: Supplementary file 15 — Reporting Summary [file 42003_2022_4027_MOESM15_ESM.pdf]

nature portfolio

Corresponding author(s): Yong Wang

Last updated by author(s): Aug 19, 2022

Reporting Summary

Nature Portfolio wishes to improve the reproducibility of the work that we publish. This form provides structure for consistency and transparency in reporting. For further information on Nature Portfolio policies, see our Editorial Policies and the Editorial Policy Checklist.

Statistics

For all statistical analyses, confirm that the following items are present in the figure legend, table legend, main text, or Methods section.

n/a	Confirmed	
☐	☒	The exact sample size (*n*) for each experimental group/condition, given as a discrete number and unit of measurement
☐	☒	A statement on whether measurements were taken from distinct samples or whether the same sample was measured repeatedly
☐	☒	The statistical test(s) used AND whether they are one- or two-sided<br>*Only common tests should be described solely by name; describe more complex techniques in the Methods section.*
☒	☐	A description of all covariates tested
☒	☐	A description of any assumptions or corrections, such as tests of normality and adjustment for multiple comparisons
☐	☒	A full description of the statistical parameters including central tendency (e.g. means) or other basic estimates (e.g. regression coefficient) AND variation (e.g. standard deviation) or associated estimates of uncertainty (e.g. confidence intervals)
☐	☒	For null hypothesis testing, the test statistic (e.g. *F*, *t*, *r*) with confidence intervals, effect sizes, degrees of freedom and *P* value noted<br>*Give P values as exact values whenever suitable.*
☒	☐	For Bayesian analysis, information on the choice of priors and Markov chain Monte Carlo settings
☒	☐	For hierarchical and complex designs, identification of the appropriate level for tests and full reporting of outcomes
☐	☒	Estimates of effect sizes (e.g. Cohen's *d*, Pearson's *r*), indicating how they were calculated

Our web collection on statistics for biologists contains articles on many of the points above.

Software and code

Policy information about availability of computer code

Data collection	Bowtie2 v2.4.1, SPAdes v.3.13, fastp v.0.2.0,SortMeRNA (v.2.1)
Data analysis	What the Phage v.1.0.1, Checkv v.0.8.1, MAFFT v7.453, trimAl v1.4.rev15, IQ-TREE v2.0.3, iTOL v.4, BWA v.0.7.17, samtools v.1.9, CoverM v0.6.1, R v.4.0.4, vegan v.2.5-7, ggpubr v.0.4.0, pheatmap v.1.0.12, Prodigal v.2.6.3, eggNOG-mapper v.2.0.0, BLASTn v2.9.0+, DRAM-v v.1.2.4, VIBRANT v.1.2.1, Phyre v.2, crass v1.0.1, , MinCED v.0.4.2, tRNA-scan v. 2.0.7, VirMatcher, WIsH, vConTACT2 v.0.9.19, Vegan v2.5-7, Virsorter2 v.2.2.3, MARVEL v.0.2, DeepVirFinder v.1.0, blastp (v.2.9.0+), iTOL v4, SMART

For manuscripts utilizing custom algorithms or software that are central to the research but not yet described in published literature, software must be made available to editors and reviewers. We strongly encourage code deposition in a community repository (e.g. GitHub). See the Nature Portfolio guidelines for submitting code & software for further information.

Data

Policy information about availability of data

All manuscripts must include a data availability statement. This statement should provide the following information, where applicable:

- Accession codes, unique identifiers, or web links for publicly available datasets
- A description of any restrictions on data availability
- For clinical datasets or third party data, please ensure that the statement adheres to our policy

All software and R packages used are open source and described in the Methods section. No custom code was used to analyze data in this study, and further details are available on request.

Human research participants

Policy information about studies involving human research participants and Sex and Gender in Research.

Reporting on sex and gender	N/A
Population characteristics	N/A
Recruitment	N/A
Ethics oversight	N/A

Note that full information on the approval of the study protocol must also be provided in the manuscript.

Field-specific reporting

Please select the one below that is the best fit for your research. If you are not sure, read the appropriate sections before making your selection.

☒ Life sciences ☐ Behavioural & social sciences ☐ Ecological, evolutionary & environmental sciences

For a reference copy of the document with all sections, see nature.com/documents/nr-reporting-summary-flat.pdf

Life sciences study design

All studies must disclose on these points even when the disclosure is negative.

Sample size	Thirteen sediment cores were collected from both slope and bottom-axis sites in Challenger Deep at depths ranging from 5,400 m to 10,911 m. The north and south slope samples were collected at every ~1000m depth. Hence, our samples cover all depths of the both slopes of the Challenger Deep. We also obtained four replicates from different sites in the bottom-axis sites. From the sediment cores, we randomly select 2-4 layers from oxic and anoxic layers. Therefore, 37 subsamples were used for metagenome analysis. The 3 subsamples from one bottom-axis core were used for metatranscriptome analysis.
Data exclusions	No data were excluded from this manuscript.
Replication	According to the data and methods provided in the papers, all results can be reproduced.
Randomization	All sediment cores were used to represent both slope and bottom-axis sites in Challenger Deep and water depth profile. Subsamples of sediment cores by 2- or 3-centimeter layers were selected to represent sediment depth profile. We randomly selected 2-4 layers from oxic and anoxic layers from similar layer depths. For samples in each layer, they were homogenized for various treatments.
Blinding	Blinding was not applicable to this study. All methods and results that we used for analysis sediment samples are objective.

Reporting for specific materials, systems and methods

We require information from authors about some types of materials, experimental systems and methods used in many studies. Here, indicate whether each material, system or method listed is relevant to your study. If you are not sure if a list item applies to your research, read the appropriate section before selecting a response.

Materials & experimental systems

n/a	Involved in the study	
☒	☐	Antibodies
☒	☐	Eukaryotic cell lines
☒	☐	Palaeontology and archaeology
☒	☐	Animals and other organisms
☒	☐	Clinical data
☒	☐	Dual use research of concern

Methods

n/a	Involved in the study	
☒	☐	ChIP-seq
☒	☐	Flow cytometry
☒	☐	MRI-based neuroimaging
